# Propofol-Related Infusion Syndrome in a Child With Refractory Status Epilepticus: Successful Resuscitation With Veno-Arterial Extracorporeal Membrane Oxygenation, Continuous Renal Replacement Therapy, and Therapeutic Plasma Exchange

**DOI:** 10.7759/cureus.47866

**Published:** 2023-10-28

**Authors:** Joshua Calvano, Matthew R Paluska, Arthur J Armijo, Timothy R Petersen, Codruta Soneru, Alia Broman, Gloria Lopez-Hernandez

**Affiliations:** 1 Department of Anesthesiology and Critical Care, University of New Mexico School of Medicine, Albuquerque, USA; 2 Department of Anesthesiology, Rocky Vista University College of Osteopathic Medicine, Englewood, USA; 3 Department of Graduate Medical Education, Healthcare Corporation of America/HealthOne, Lone Tree, USA; 4 Department of Graduate Medical Education, University of New Mexico School of Medicine, Albuquerque, USA; 5 Department of Obstetrics and Gynecology, University of New Mexico School of Medicine, Albuquerque, USA; 6 Department of Pediatrics, University of Colorado School of Medicine, Denver Health Medical Center, Denver, USA; 7 Department of Pediatrics, University of New Mexico School of Medicine, Albuquerque, USA

**Keywords:** continuous renal replacement therapy (crrt), propofol infusion syndrome, refractory status epilepticus, therapeutic plasma exchange (tpe), va-ecmo

## Abstract

Propofol is used for sedation, anxiolysis, anesthesia induction, and as an anticonvulsant. In cases of refractory status epilepticus (RSE), propofol is more efficient than barbiturates. We present a case of a 3-year-old female with RSE who developed propofol-related infusion syndrome (PRIS) despite low dosage after failed attempts with multiple anti-epileptic drips and bolus therapies. Careful consideration must be made before initiating propofol administration for RSE. We discuss our PRIS treatment approach with extracorporeal membrane oxygenation, therapeutic plasma exchange, and continuous renal replacement therapy leading to our patient recovering to baseline and being discharged home from the hospital.

## Introduction

Propofol (2,6-diisopropylphenol) is an intravenous medication advantageous in critical care and operative settings due to its rapid onset and short duration of action. It is commonly used for sedation, anxiolysis, induction of anesthesia, and anticonvulsant properties [1]. In cases of refractory status epilepticus (RSE), propofol has been shown to be more efficient than barbiturates for prompt control and shortens average tracheal intubation placement time [2].

However, despite efficacy in RSE management, critically ill patients who require long duration (>48 hours) or high doses (≥5 mg·kg^-1^·hr^-1^) of propofol-infusion are at high risk for propofol-related infusion syndrome (PRIS) [3]. The development of PRIS is currently believed to be influenced by the combination of propofol-induced biochemical alterations and the individual's current health condition (e.g., sepsis, shock, trauma, etc.) along with the simultaneous use of other drugs [4]. Moreover, there is emerging evidence suggesting a link between PRIS and mitochondrial defects, raising concerns that propofol may exacerbate or even unveil underlying mitochondrial disorders in some patients [5]. Propofol is thought to suppress the function of carnitine palmitoyltransferase I, an enzyme responsible for attaching a fatty acyl group to carnitine [4]. When this enzyme is less active it disrupts the beta-oxidation process of fatty acids [6]. This disruption allows for an energy disparity, typically during a critical illness state, that is thought to explain the myocytolysis of skeletal and cardiac muscles in PRIS [6]. The buildup of fatty acids in organs has been associated with the onset of cardiac arrhythmias [4]. This arrhythmogenicity is further exacerbated by propofol's calcium channel-blocking properties, which can reduce cardiac output [1].

Incidents of PRIS pediatric cases have been documented since the first reported death of a Danish 3-year-old in 1990 [7]. PRIS symptomatology varies, but commonly documented system disorders include cardiac (cardiac failure, including pulmonary edema, widened QRS, bradycardia, ventricular tachycardia or fibrillation, asystole), vascular (hypotension), renal (acute kidney injury or AKI and urine color changes), musculoskeletal (rhabdomyolysis), metabolic (metabolic acidosis, hyperkalemia, lipidemia), and hepatobiliary (hepatomegaly and elevated liver transaminases) without other reasonable explanation [3].

The case presented describes a 3-year-old patient with RSE, requiring control with propofol, who developed PRIS after 24 hours resulting in 3 cardiopulmonary arrests. This case demonstrates the importance of PRIS recognition. It describes a multi-system aggressive treatment approach utilizing veno-arterial extracorporeal membrane oxygenation (VA-ECMO), continuous renal replacement therapy (CRRT), and therapeutic plasma exchange (TPE).

Written Health Insurance Portability and Accountability Act authorization has been obtained from the patient's parents for the publication of this case report. This manuscript adheres to the applicable Enhancing the Quality and Transparency of Health Research Network (EQUATOR) guidelines using the Case Report (CARE) checklist.

## Case presentation

Our patient was a 3-year-old (13 kg) female with a past medical history of left hemiparetic (Gross Motor Function Classification System 4-5) cerebral palsy, cerebral visual impairment, bronchopulmonary dysplasia with a tracheostomy tube and gastric-tube dependency, and focal epilepsy who presented in status epilepticus.

The patient's seizures had previously been well controlled, and she was seizure-free for over 1 year until 5 months prior to admission, when she began having seizures roughly once weekly. Seizure semiology ranged from absence to partial focal to generalized tonic-clonic without apparent preference for any particular type. The patient had been placed on a ketogenic diet 4 weeks prior and was continuing neurology-prescribed home medications. For 2 weeks prior to admission, the patient had increased seizures (1-2 per day), typically absence, described as staring spells with unresponsiveness and no postictal period. Video electroencephalogram (vEEG) in the emergency room demonstrated status epilepticus. She was admitted to the pediatric intensive care unit (PICU).

She remained in RSE despite multiple seizure medication drips including midazolam, pentobarbital, and ketamine. She additionally received push doses of valproate and fosphenytoin before a decision was made by her care teams to start a propofol infusion to achieve burst suppression. Burst suppression occurred with slow up-titration of propofol infusion to a maximum rate of 258 μg·kg^-1^·hr^-1^ and ketamine of 3 mg·kg^-1^·hr^-1^. After 24 hours on propofol, a wean was started. However, it was abruptly discontinued as the patient developed signs and symptoms consistent with PRIS (AKI, rhabdomyolysis, transaminitis, triglyceridemia, lactic acidosis, and bradyarrhythmias with significant hypotension). Midazolam drip was restarted after propofol discontinuation, but vEEG showed a return of seizures, so valproate bolus was administered, and a drip was started for suppression.

**Figure 1:**
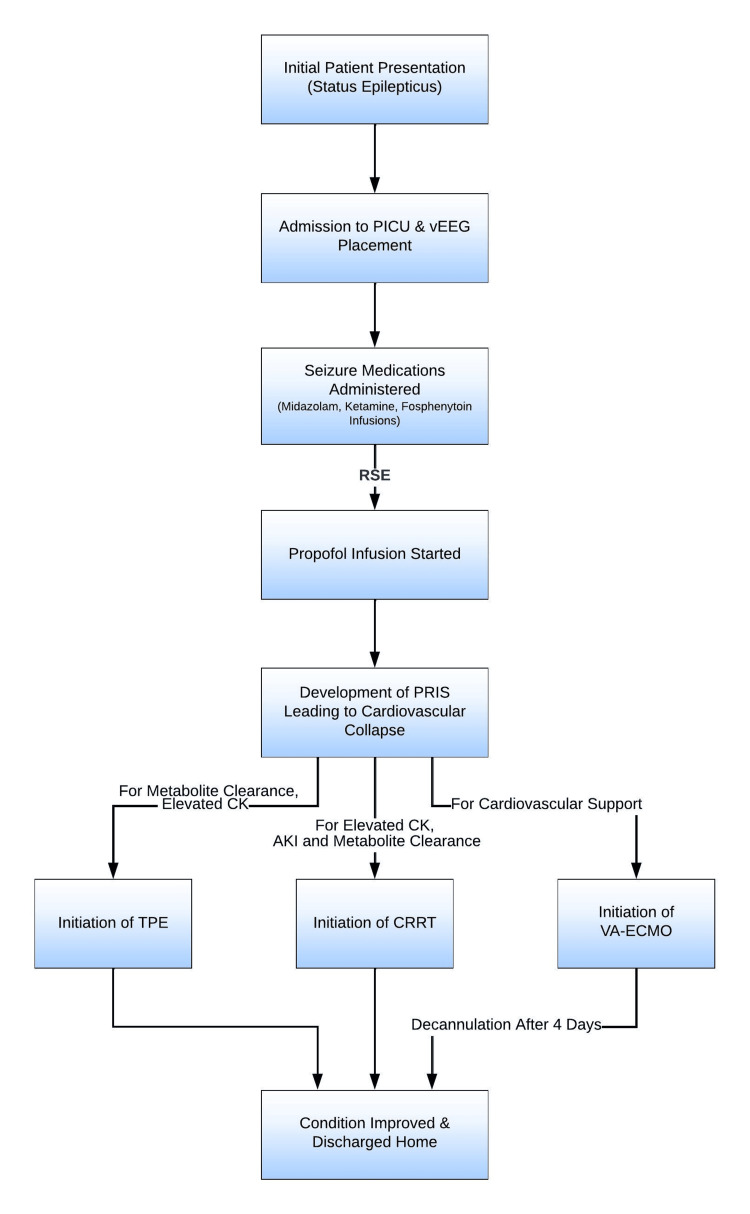
Clinical management and treatment pathway for our 3-year-old female patient presenting with refractory status epilepticus and subsequent development of PRIS. The pathway incorporates interventions for cardiovascular and metabolic complications arising from PRIS. Critical interventions include VA-ECMO for cardiovascular support, CRRT for elevated CK plus AKI and metabolite clearance, and TPE for elevated CK levels plus metabolite clearance. PRIS: Propofol-Related Infusion Syndrome, PICU: Pediatric Intensive Care Unit, vEEG: Video Electroencephalogram, AKI: Acute Kidney Injury, CK: Creatinine Kinase, VA-ECMO: Veno-Arterial Extracorporeal Membrane Oxygenation, CRRT: Continuous Renal Replacement Therapy, TPE: Therapeutic Plasma Exchange

During the first evening following the suspected PRIS, she had 3 total code events requiring code dose medications and 1 round of cardiopulmonary resuscitation. Initially, a Vas-Cath® was inserted into the right internal jugular vein to initiate CRRT for her AKI. Subsequently, following a multi-disciplinary decision to commence VA-ECMO, the same right internal jugular access site was utilized, along with the right carotid artery. The CRRT was run through the ECMO circuit so no new access was needed. Due to complications with the tracheostomy ties, her tracheostomy tube was removed and she was subsequently orally intubated to facilitate the cannulation process. Pediatric surgery cannulated her at the bedside, and she tolerated the procedure well. In addition to CRRT, TPE was added to remove harmful propofol metabolites and to help correct her severely elevated creatinine kinase levels (Figure 1). She continued to have persistent rhabdomyolysis but became more cardiovascularly stable and had an uneventful decannulation 4 days later.

## Discussion

Status epilepticus is a seizure with 5 or more minutes of seizure activity without recovery defined by electroencephalogram or clinical symptoms [8]. When 3 intravenous drugs fail to terminate the seizure, this is termed refractory status epilepticus (9-31% of status epilepticus [9]), with a mortality rate of 35% [8]. When first-line medications (e.g., phenytoin) fail, barbiturates, benzodiazepines, ketamine, and propofol have been used [8]. Our case demonstrates that although propofol remains a viable option for controlling RSE, physicians must weigh the potential benefits against the risks, especially in pediatric patients. Using a high infusion rate or administering a bolus of propofol can elevate the risk of PRIS [6].

Although our patient received propofol infusions well below the usual threshold of 5 mg·kg^-1^·hr^-1^ and a duration of <48 hours, she developed PRIS [3]. While this threshold appears to be a common concept, Svensson, and Lindberg found no relation between doses of >3 mg·kg^-1^·hr^-1^ or ≥48 hours of propofol infusion duration causing PRIS [10]. These findings suggest that PRIS development is multifactorial and still not fully understood. Likewise, target lab values for monitoring PRIS evolution in critically ill patients are unclear. However, lactic acidosis, renal failure, and rhabdomyolysis are typical early signs [11]. These early signs were seen in our case, along with transaminitis, triglyceridemia, and bradyarrhythmias with significant hypotension within 24 hours of starting the propofol infusion.

A 2015 case study first described TPE’s use in PRIS, producing immediate results of improved hemodynamics and resolution of lactic acidosis within 24 hours [12]. TPE involves separating and discarding plasma from blood cells and replacing it with donated plasma or albumin [12]. TPE's main risk is exposure to fresh frozen plasma, a potential allergen [12]. The procedure may mitigate PRIS by removing excess triglycerides bound to propofol (Table 1) [12]. Furthermore, TPE can remove excess long-chain fatty acids involved in the pathophysiology of PRIS. Therefore, TPE should theoretically reduce the risk of arrhythmogenicity and mortality in PRIS by decreasing the likelihood of cardiac failure and therapy-resistant bradyarrhythmias (Table 1) [12].

**Table 1:** Overview of treatment modalities, mechanisms, and proposed mechanisms in the management of propofol-related infusion syndrome PRIS: Propofol-Related Infusion Syndrome, TPE: Therapeutic Plasma Exchange, CRRT: Continuous Renal Replacement Therapy, VA-ECMO: Veno-Arterial Extracorporeal Membrane Oxygenation [12-14]

Treatment Modality	Mechanism of Modality	Mechanism of PRIS Treatment
Therapeutic Plasma Exchange (TPE)	Plasma is separated from the patient’s blood and then replaced with fresh frozen plasma or another replacement solution	Removes excess triglycerides and long-chain fatty acids thought to reduce bound propofol and reduce arrhythmogenicity
Continuous Renal Replacement Therapy (CRRT)	Continuous dialysis to allow solute and fluid homeostasis	Removal of toxic water-soluble metabolites of propofol
Veno-Arterial Extracorporeal Membrane Oxygenation (VA-ECMO)	Drawing deoxygenated blood out of the venous side, oxygenating it externally, returning oxygenated blood to arteries	Myocardial recovery due to decreased cardiac and respiratory work

CRRT is an effective solution for managing rhabdomyolysis due to PRIS. Honore and Spapen recommend initiating CRRT early in PRIS, even without typical clinical signs like AKI, metabolic acidosis, or elevated potassium levels [13]. The recommendation is based on propofol metabolism. The liver metabolizes propofol to propofol-glucuronide and other conjugates, which the kidneys expel [13]. Unlike lipophilic propofol, these metabolites are water-soluble and toxic (Table 1) [13].

To the authors’ best knowledge, only 3 additional cases of VA-ECMO use in PRIS have been published discussing the role of VA-ECMO as a treatment option for PRIS in the pediatric population [6]. The role of VA-ECMO in PRIS treatment allows for cardiac recovery, typically within days of cannulation. Although not used in this case, other case reports have suggested ventricular assist devices for myocardial recovery if VA-ECMO is insufficient (Table 1) [14].

## Conclusions

The main consistent point in the literature is that close monitoring during propofol infusion is essential. Upon concern of PRIS, propofol should promptly be discontinued and multiple treatment modalities including TPE, CRRT, and VA-ECMO should be considered if severe, especially if there are no contraindications. Regardless, our case demonstrates that prompt recognition of PRIS is vital to starting treatment as soon as possible.

To the authors' best knowledge, this case study is the first to discuss the concurrent use of all 3 of the therapeutic modalities described above for treating PRIS in pediatric patients. In the setting of cardiovascular collapse, we recommend early initiation of VA-ECMO as a potentially lifesaving measure. The literature on VA-ECMO therapy for PRIS treatment in pediatrics remains limited, with only a few cases described. Additional therapies including CRRT and TPE should be considered for correcting lab abnormalities and clearing propofol metabolites. Future studies are needed to understand further the risks and benefits of this therapy combination. We believe that our multimodal treatment approach to PRIS in this pediatric patient positioned her well for recovery to baseline and discharge from the hospital.
